# Secondary Syphilis Presents as Palmoplantar Hyperpigmented Maculopapules: A Case Report

**DOI:** 10.7759/cureus.57367

**Published:** 2024-04-01

**Authors:** Muhjah M Almurakshi, Bushra A Fatani, Ahmed Niyazi, Ahmed H Alajlan, Marwan Alzahrani, Nada Fatani, Hadeel Alabdali, Khalid Al Hawsawi

**Affiliations:** 1 Medicine, Umm Al-Qura University, Makkah, SAU; 2 Medicine, King Saud University, Riyadh, SAU; 3 Medicine, Ibn Sina National College for Medical Studies, Jeddah, SAU; 4 Infectious Disease, King Abdulaziz Hospital, Makkah, SAU; 5 Internal Medicine, King Abdulaziz Hospital, Makkah, SAU; 6 Dermatology, King Abdulaziz Hospital, Makkah, SAU

**Keywords:** treponema pallidum, hiv, condyloma lata, leus, secondary syphilis

## Abstract

Syphilis is a worldwide chronic systemic sexually transmitted infection caused by the spirochete bacterium *Treponema pallidum*. Here, we report a 28-year-old homosexual male who presented to the dermatology clinic with a six-month history of asymptomatic persistent skin lesions. A review of systems revealed unintentional weight loss of about 40 kg within one year. Skin examination revealed multiple scaly and non-scaly hyperpigmented macules and patches on the palms and soles. Hair, nail, and mucus membrane examinations were normal. There was no lymphadenopathy. A skin biopsy revealed psoriasiform acanthosis, lichenoid infiltrates with moderately dense mononuclear lymphohistiocytic cells, few plasma cells, and eosinophils. Laboratory investigations revealed positive rapid plasma reagin (RPR) with a titer of 1:128. *Treponema* *pallidum* hemagglutination test (TPHA) was positive. The HIV test by western blot was positive. Based on the above clinicopathological and laboratory findings, a diagnosis of secondary syphilis was made in this patient, who also tested positive for HIV. He was given a single dose of penicillin G benzathine (2.4 units) intramuscularly. He was also started on Dolutegravir 50 mg tablet once daily and Tenofovir alafenamide fumarate + Emtricitabine tablet once daily. Three months after penicillin G benzathine treatment, the RPR test turned negative, and the skin lesions disappeared.

## Introduction

Syphilis is a worldwide chronic systemic sexually transmitted infection caused by the spirochete bacterium *Treponema pallidum *[1]. The prevalence varies significantly across different geographical regions [1]. The highest risk group is men who have sex with men [1]. Syphilis passes through active and latent stages (LS) [2]. The active dynamic stages include primary, secondary, and tertiary [2]. The LS is the period between the healing of the secondary stage and the appearance of tertiary syphilis [2]. The LS is characterized by positive serologic tests for specific antibodies without clinical signs or symptoms [2]. The LS is classified as either early latent (less than one year) or late latent (more than one year) [2]. Seventy percent of untreated individuals will remain in this stage for the rest of their lives and are immune to new primary infections [2]. Early syphilis includes primary, secondary, and early latent, while late syphilis has late latent and tertiary stages. The systemic manifestations can be seen in these three stages but are rare in the primary stage. The infectious stage is early syphilis [3]. The secondary stage appears either simultaneously with the preliminary stage or up to six months after the healing of the primary stage [3]. The prodromal symptoms of the secondary stage include low-grade fever, malaise, sore throat, lymphadenopathy, weight loss, muscle aches, and headache [3]. It lasts several weeks to months, with a 25% relapse during the latent stage [4].

## Case presentation

A 28-year-old homosexual male presented to the Dermatology clinic with a six-month history of asymptomatic persistent skin lesions. A review of systems revealed unintentional weight loss of about 40 kg within one year. There was no history of fever, fatigue, genital ulcer, headache, hearing loss, neurological symptoms, or eye symptoms. The patient had no significant past medical history, surgical procedures, or drug history, and there was no family history of a similar condition.

**Figure 1:**
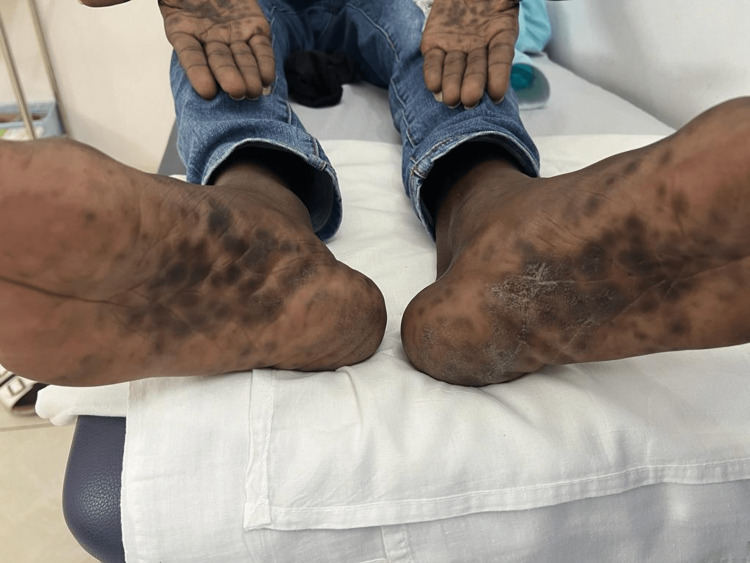
The patient's palms and soles show multiple non-scaly hyperpigmented macules and patches.

**Figure 2:**
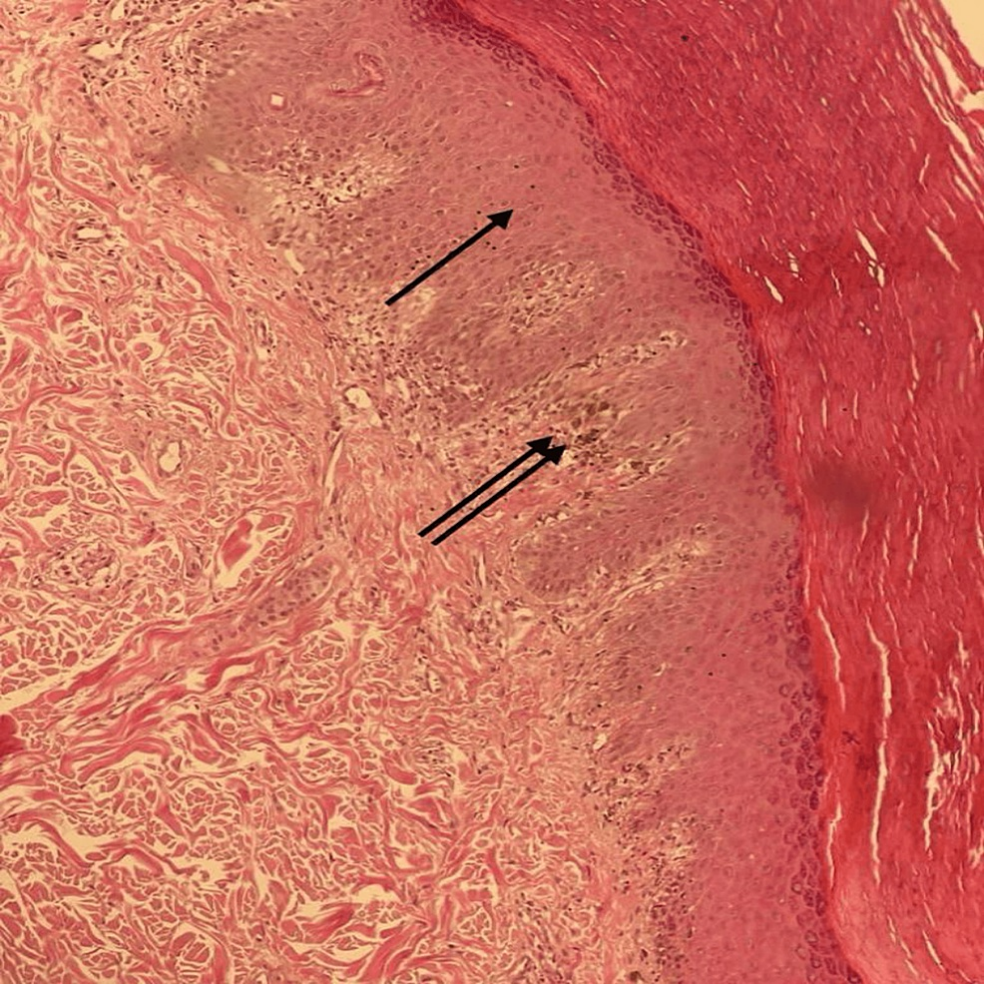
Skin biopsy revealed psoriasiform acanthosis (single arrow), lichenoid infiltrates with moderately dense mononuclear lymphohistiocytic cells, few plasma cells, and eosinophils (double arrow).

Skin examination revealed multiple scaly and non-scaly hyperpigmented maculopapules on the palms and soles (Figure 1). There were no other skin lesions. Hair, nail, and mucus membrane examinations were normal. There was no lymphadenopathy. Skin biopsy revealed psoriasiform acanthosis, lichenoid infiltrates with moderately dense mononuclear lymphohistiocytic cells, few plasma cells, and eosinophils (Figure 2).

**Figure 3:**
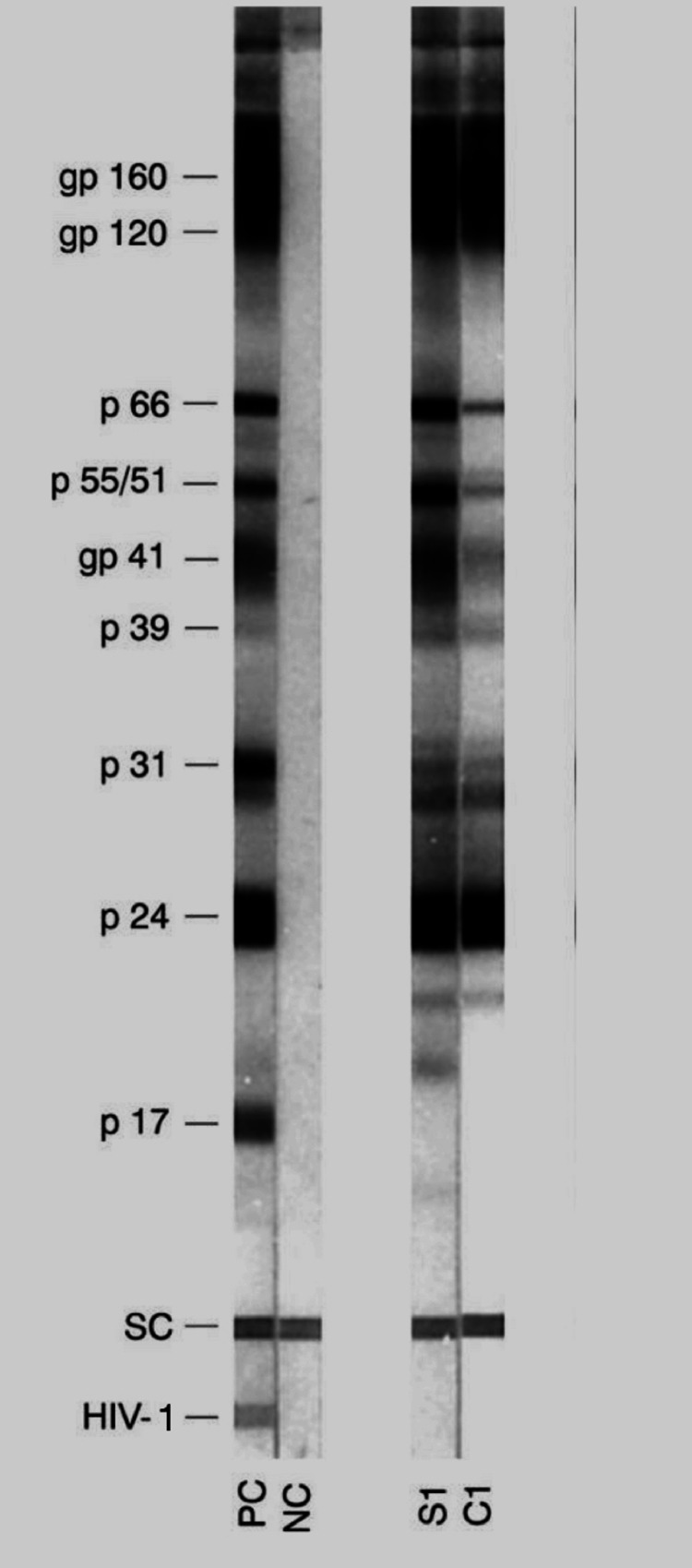
Western blot test shows positive HIV-1 test. PC: positive control; NC: negative control; S1: Sample 1; C1: Control 1.

Laboratory findings revealed normal levels of CBC (Table 1), liver panel (Table 2), and renal panel (Table 3). The HIV test by enzyme-linked immunosorbent assay (ELISA) and western blot was positive (Figure 3). The HIV-1 assay was 72.743 copies/mL. Rapid plasma reagin (RPR) was positive with a titer of 1:128. *Treponema pallidum* hemagglutination test (TPHA) was positive (reference negative).

**Table 1:** CBC with differential.

Procedures	Result	Unit	Reference range
White blood cells	6.44	10^9^/L	4-11
Red blood cells	5.42	10^12^/L	4.5-6
Hemoglobin	168	g/L	130-180
Hematocrit	0.496	L/L	0.4-0.52
Mean corpuscular volume	91.5	FL	80-96
Mean corpuscular hemoglobin	31	Pg	27-32
Mean corpuscular hemoglobin concentration	339	g/L	300-350
Platelet count	202	10^9^/L	150-450
Neutrophil %	27	%	40-75
Lymphocytes%	52.5	%	20-45
Monocytes %	10.9	%	2-10
Eosinophil %	8.5	%	1-6
Basophils %	1.1	%	0-1
Neutrophil count	1.74	10^9^/L	2-7.5
Lymphocytes count	3.38	10^9^/L	1-4.8
Monocytes count	0.7	10^9^/L	0.2-1
Eosinophil count	0.55	10^9^/L	0.04-0.5
Basophils count	0.07	10^9^/L	0-0.1

**Table 2:** Liver function test.

Procedures	Result	Unit	Reference range
Total bilirubin	10.4	Umol/L	3.4-20.5
Conjugated bilirubin	2.02	Umol/L	0-3.4
Alkaline phosphatase	59	U/L	40-150
Alanine transaminase	12	U/L	0-55
Aspartate aminotransferase	17	U/L	5-34

**Table 3:** Renal function test.

Procedures	Result	Unit	Reference range
Sodium	139	mmol/L	136-145
Potassium	4.8	mmol/L	3.5–5.1
Chloride	103	mmol/L	98-107
Urea	3.8	mmol/L	2.5–9.2
Creatinine	99.6	mmol/L	63.6–110.5

**Figure 4:**
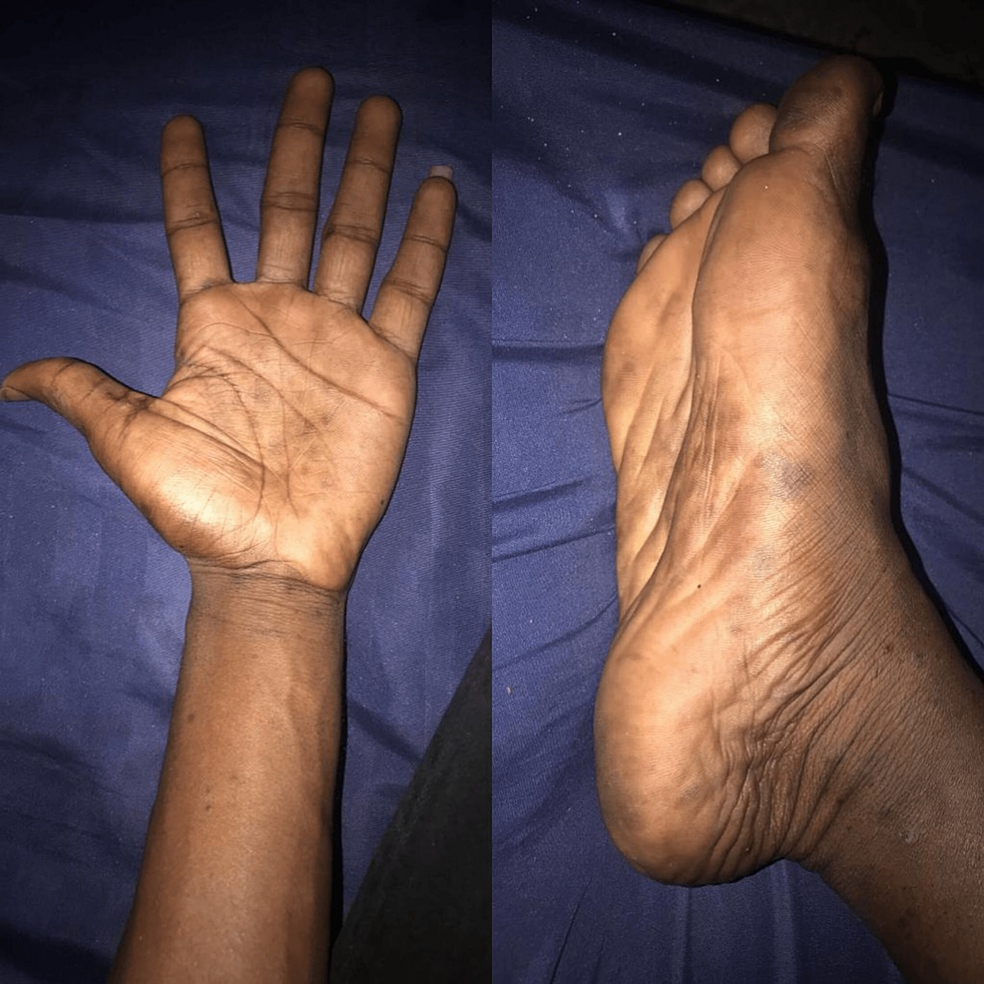
Clearance of the lesions three months after a single dose of benzathine penicillin.

Based on the clinicopathological and laboratory findings, the patient was diagnosed with secondary syphilis and HIV positive. He does not recall prior syphilis diagnosis or treatment. Public health authorities were notified about this case. He was given a single dose of penicillin G benzathine (2.4 units) intramuscularly (IM). The infectious disease internist started the patient on Dolutegravir 50 mg tablet once daily and Tenofovir alafenamide fumarate + Emtricitabine tablet once daily. The viral load after two months of beginning antiretroviral treatment was not detected. The patient was counseled to trace and treat at-risk sex partners. Three months after penicillin G benzathine treatment, the RPR test turned negative, and the skin lesions disappeared (Figure 4). The patient is under periodic follow-up with the infectious diseases clinic.

## Discussion

Secondary syphilis results from the hematogenous and lymphatic dissemination of treponemes [4]. The patterns of skin lesions in syphilis include (1) generalized roseola-like non-pruritic lesions that appear early in 10% of patients; (2) generalized maculopapular and papulosquamous eruptions with more infiltrated lesions, often copper-colored, annular plaques on the face, lesions with the corymbose arrangement (satellite papules around a more significant central lesion) [4]; and (3) localized lesions, which can be (i) palmoplantar, symmetrical papules, and plaques with a scale (collarette of Biett); (ii) anogenital (condylomata lata); and (iii) seborrheic area: “corona veneris” along the scalp's hairline [5].

The most reliable dermatoscopic finding of secondary syphilis lesions is the presence of a diffuse orangish or yellowish-red background with vascularity [6]. The hypopigmented lesions can be generalized or localized mainly on the neck, known as the “necklace of Venus" [5]. Additional findings include non-scarring “moth-eaten” alopecia, split papules at the oral commissures, granulomatous nodules and plaques, crusted necrotic lesions, and malignant syphilis known as lues maligna (disseminated ulcers), which is extremely rare [7]. Our case showed non-scaly hyperpigmented palmoplantar macules and patches rather than papules and plaques.

Syphilis facilitates the transmission of HIV [8]. Syphilis produces genital ulcers that increase the risk of acquiring HIV [8]. Many factors contribute to the increased risk of HIV transmission, including the lack of an epithelial barrier due to skin ulceration, large numbers of macrophages and T cells with receptors for HIV, and an increase in cytokines produced by macrophages stimulated by treponemal lipoproteins [8]. Syphilis manifestations are altered in HIV-positive patients, with higher neurologic manifestations and ulcerative lesions (in secondary syphilis) [8]. However, our patient did not show any of these. Although non-treponemal antibody tests (RPR or venereal disease research laboratory (VDRL)) can be temporarily negative in HIV-positive patients, this was not seen in our patients [8]. The natural history of untreated syphilis will be as follows: the skin lesions of primary and secondary stages spontaneously disappear, the treponemal antibody test (e.g., microhemagglutination assay for *Treponema pallidum* antibodies (MHA-TP)) remains positive for life, and the non-treponemal antibody test is negative in one-third of patients and stays positive in two-thirds of patients (one-third of patients develop tertiary syphilis and one-third of patients are asymptomatic) [8]. In syphilis, whether treated or untreated, treponemal antibody tests remain positive for life, except in the case of treatment of very early syphilis, where 25% of patients revert to negative. In treated syphilis, non-treponemal antibody tests revert negative (except in a few cases, which may remain positive for life) [8].

An analysis of the cerebrospinal fluid (CSF) following a lumbar puncture must be performed in patients with ocular or neurologic manifestations or when treatment failure is suspected (e.g., persistent/recurrent signs/symptoms or failure of non-treponemal test titers to decline fourfold within 6-12 months) [9]. However, none of these were present in our patient.

Treatment of early syphilis includes benzathine penicillin (2.4 million units of single-dose IM) or procaine penicillin (1.2 million units IM) daily for 10 days [9]. Treatment of penicillin-allergic patients includes doxycycline 200 mg daily (100 mg PO BID preferred over a single 200 mg dose) for 14 days or tetracycline 500 mg PO four times daily for 14 days, ceftriaxone 1-2 g IM or IV daily for 10-14 days, or azithromycin 2 g PO as a single dose [9]. However, bacterial resistance has been reported with azithromycin.

The skin lesions in our patient disappeared after three months of post-penicillin G benzathine treatment. However, the lesions can resolve spontaneously over several weeks even without treatment [9]. The RPR test is also used to monitor the effectiveness of a treatment [10]. There should be a fourfold decrease in RPR titer within 6-12 months to consider the successful treatment [10]. However, the RPR test in our patient turned negative three months after penicillin G benzathine treatment.

HIV-positive patients are at increased risk of neurosyphilis, especially if they have a CD4 count of <350 cells/mL and an RPR titer of ≥1 32 [10]. Therefore, careful follow-up is mandatory for monitoring ocular or neurologic manifestations [10].

The International Union Against Sexually Transmitted Infection (IUSTI) recommends that non-treponemal tests (e.g., VDRL, RPR) be performed one, two, three, and six months following antibiotic treatment for early syphilis, followed by every six months for up to two years post-treatment. The CDC suggests clinical and serologic evaluation for uncomplicated cases at six and 12 months, with more frequent and extended assessment (e.g., at three, six, nine, 12, and 24 months) for HIV-infected patients [10]. Careful follow-up is mandatory for the early detection of relapse or reinfection. Retreatment is compulsory if there is a fourfold increase in titer [11].

The patient was counseled to trace and treat at-risk sex partners. Identifying which sex partners are at risk of infection depends on the elapsed time since last exposure and the stage of disease in the source patient. The risk period is three months plus the duration of symptoms for primary syphilis, six months plus the duration for secondary syphilis, and one year plus the duration of symptoms for latent syphilis. Management of at-risk sex partners depends on the elapsed time since exposure. Sex partners exposed during the three months preceding a diagnosis of primary, secondary, or latent syphilis should be tested for syphilis [11]. However, regardless of the results, those partners should be treated because of the high efficacy of the treatment and the likelihood that they have been infected but have not yet shown clinical or lab evidence of the disease [11]. Sexual partners of persons with late latent syphilis should be evaluated, tested, and managed accordingly [11].

## Conclusions

Since HIV infection alters the immune system, clinical symptoms in secondary syphilis patients with HIV are atypical and overlap. The spread of the syphilis virus is accelerated by unsafe sexual behavior among men who engage in homosexual sex. Therapy and diagnosis are typically the same. Administration of a benzathine penicillin injection once gave lesion improvement and significantly reduced VDRL.
